# Correlation Between Ultra‐High Frequency Ultrasound (UHFUS) and Histological Features of Typical Lesions in Hidradenitis Suppurativa

**DOI:** 10.1002/jum.16759

**Published:** 2025-06-28

**Authors:** Alessandra Michelucci, Giammarco Granieri, Cristian Scatena, Bianca Cei, Flavia Manzo Margiotta, Teresa Oranges, Marco Romanelli, Valentina Dini

**Affiliations:** ^1^ Department of Dermatology University of Pisa Pisa Italy; ^2^ Interdisciplinary Center of Health Science Sant'Anna School of Advanced Studies of Pisa Pisa Italy; ^3^ Division of Pathology, Department of Translation Research on New Technologies in Medicine and Surgery University of Pisa Pisa Italy; ^4^ Department of Laboratory Medicine Pisa University Hospital, Anatomia Patologica 1 Universitaria Pisa Italy; ^5^ Dermatology Unit Meyer Children's Hospital, IRCCS Florence Italy

**Keywords:** hidradenitis suppurativa, histology, tunnel, UHFUS

## Abstract

**Objectives:**

Hidradenitis suppurativa (HS) is a chronic inflammatory disease affecting hair follicles, significantly impacting patients' quality of life. While clinical assessment remains the cornerstone of diagnosis and staging, ultrasound (US) has emerged as a valuable tool for disease evaluation, treatment guidance, and monitoring. The introduction of ultra‐high frequency ultrasound (UHFUS) allowed for near in vivo histopathological examination of HS lesions. This study aimed to correlate UHFUS features with histopathological findings in HS lesions.

**Methods:**

Three patients at different disease stages underwent pre‐surgical UHFUS mapping using a 70 MHz linear probe. Regions of interest (ROIs) were identified and marked preoperatively. Patient 1 had an abscess and perilesional skin, Patient 2 had a draining abscess, and Patient 3 had a clinically evident tunnel. Local excision was performed for Patient 1, while wide excision was conducted for Patients 2 and 3. Tissue samples underwent histological examination, and UHFUS findings were analyzed by three independent observers in consultation with a pathology expert.

**Results:**

UHFUS demonstrated the ability to delineate fluid collections, tunnels, and subclinical lesions with high precision. Case 1 revealed a fluid collection with an intra‐lesional drop‐shaped inclusion, corresponding histologically to a neutrophilic abscess with keratin‐filled cysts. Case 2 showed an elongated inclusion within a fluid collection, which histology confirmed as a keratin cyst linked to a tunnel. Case 3 exhibited a tunnel structure characterized by hyperechoic bands on UHFUS, correlating histologically with a keratinized epithelial‐lined tunnel.

**Conclusions:**

These findings highlight UHFUS as a non‐invasive tool enhancing HS assessment, bridging clinical and histopathological evaluation. Further research is needed to standardize parameters and optimize its clinical utility.

**Abbreviations**
HS: hidradenitis suppurativa
ROI: region of interest
SOS‐HS: clinical sonographic score capable of staging disease severity
US: ultrasound
UHFUS: ultra‐high frequency ultrasound

Hidradenitis suppurativa (HS) is a chronic inflammatory disease affecting hair follicles, significantly impacting patients' quality of life.^1^

The diagnosis of HS is based on a combination of clinical and ultrasonographic signs, as highlighted in the recent international consensus on the use of ultrasound in HS.^2^, ^3^

To date, the US techniques using probes with frequencies of 18–22 MHz (axial resolution of ~100 μm) reveal several key features, including dilatation of hair follicles, changes in the dermis and epidermis with the presence of pseudocysts, fluid collections and tunnels.^4^, ^5^, ^6^ Features found on US examination with probes ranging from 7 to 18 MHz have been organized and integrated into a clinical sonographic score capable of staging disease severity (SOS‐HS).^7^

Studies indicate that the severity of HS detected via US often differs from clinical assessments, highlighting US's ability to identify early and subclinical manifestations of the disease.^8^, ^9^, ^10^

In addition to disease staging, US has also been proposed as a tool able to support therapeutic choice: assessment of tunnels edema and fibrosis has been proposed as a parameter capable of guiding the therapeutic approach and monitoring its efficacy.^11^, ^12^, ^13^, ^14^ HS tunnels were classified using high‐frequency US probes (HFUS) into different subtypes, differentiated on the basis of fibrosis scarring.^3^, ^15^ More recently, the US morphological features of normal hair follicles, hair tracts and apocrine glands have been studied using an ultra‐high frequency US (UHFUS).^16^ The use of a 70 MHz probe allows a spatial resolution of 30 μm, resulting in a kind of in vivo histopathological examination. Indeed, Oranges et al showed that UHFUS can detect microcysts, micro‐tunnels, and subtle alterations of hair follicles—changes that may represent early or subclinical stages of HS.^16^ Similarly, Wortsman et al highlighted that a 70 MHz probe can reveal key signs such as “ballooned” hair follicles and retained keratin fragments, which correlate with disease severity.^17^ By visualizing these structural details, UHFUS not only improves diagnostic accuracy but also provides insights into HS pathogenesis, potentially facilitating earlier and more targeted intervention.

However, literature reports limited studies that correlate US findings with histological parameters, although existing research supports the validity of US as a biomarker for HS. For instance, Zarchi et al in 2015 correlated clinical parameters (pain and erythema) to the US diameter of nodules, while Grand et al demonstrated a strong correlation between US measurements of epidermal thickness and dermal tunnel diameter, assessed with a 10–22 MHz probe, and histological outcomes.^18^, ^19^

Nevertheless, there is no univocal correlation between US and histological parameters so far in the literature. The introduction of UHFUS may address this gap by offering more precise and reproducible measurements for histological comparison. In addition, the identification of early HS lesions with a 70 MHz probe may provide information regarding the disease pathogenesis, which is not yet completely understood.^20^

The aim of the study was to compare UHFUS features and histopathologic morphological structures of typical HS lesions.

## Methods

We enrolled three patients at different disease stages, classified according to the Hurley staging system.

Before surgery, we conducted pre‐surgical US mapping on each patient using a 70 MHz UHFUS linear probe (VevoMD, FUJIFILM, Toronto, Canada). Excision margins were outlined with a dermographic pen, and regions of interest (ROI) were identified similarly. For Patient 1, two ROIs were identified, clinically corresponding to an abscess and the perilesional skin; for Patient 2, a ROI was identified at the site of a draining abscess; for Patient 3, a ROI was positioned at the location of a clinically evident tunnel. Patient 1 underwent local excision while Patients 2 and 3 were treated with wide excision using cold‐bladed techniques. Tissue samples were subsequently fixed in formalin, sectioned according to the demarcated lines, and subjected to histological examination (Hematoxylin and Eosin staining). No microbiological or molecular testing to test sterility were performed.

All US clips were obtained by a single operator using a great amount of gel to ensure optimal contact between the probe and the stratum corneum. Each clip was analyzed by three different operators, with correlations drawn in consultation with a pathologist with expertise in skin tissue analysis.

## Results

### Case 1

**Figure 1:**
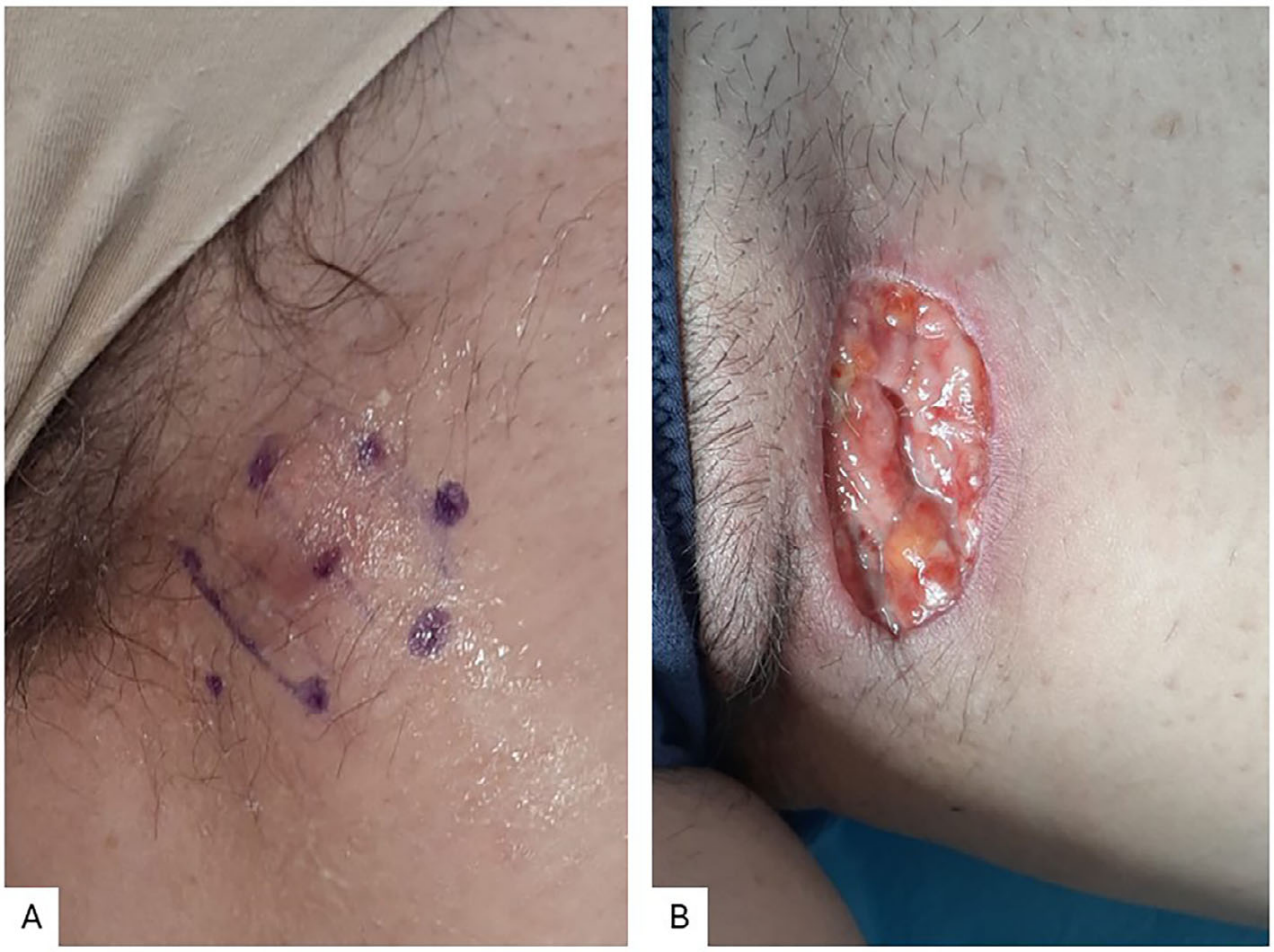
Case 1: Presurgical mapping (**A**) and postsurgical lesion after local excision of an abscess located in the left groin (**B**).

Case 1 was a 56‐year‐old female patient affected by Hurley stage I HS for 4 years, presenting with a recurrent abscess in the left groin without scarring or tunnel formation. The patient had been treated only with topical corticosteroids and antibiotics, as well as oral antibiotics as needed. The patient underwent local excision of the lesion of interest, corresponding to an abscess located in the left groin (Figure 1).

#### UHFUS Examination

**Figure 2:**
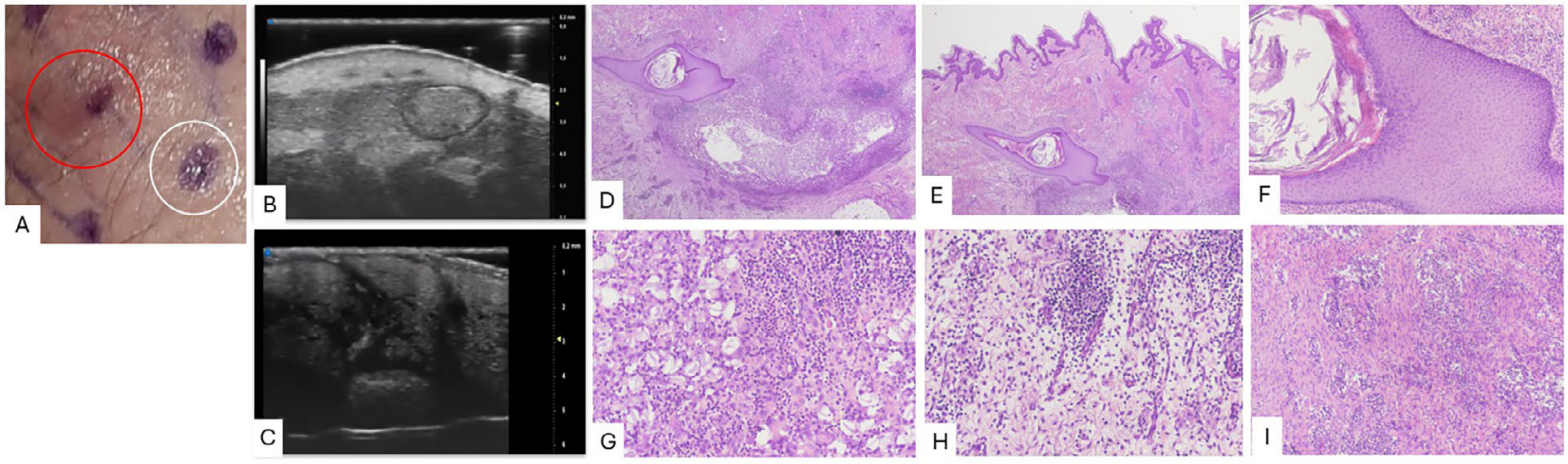
Case 1: Clinical examination of a nodule (red circle) and perilesional skin (white circle) in the left groin area (**A**). UHFUS examination reveals a single fluid collection with ballooned hair follicle or pseudocyst‐like structure (**B**), while perilesional skin reveals dilated hair follicles (**C**). Histological examination shows a dermal mixed inflammatory cell infiltrate extending into the subcutaneous tissue associated with a superficial dilated infundibulum/pseudocyst‐like structure (**D**, **E**: magnification 20×; **F**: magnification 100×). The inflammatory cell infiltrate is composed by a dense neutrophilic infiltration admixed to keratin fragments from hair shafts (**G**: magnification 200×), interstitial edema (**H**: magnification 200×), peripheral granulation tissue and fibrosis (**I**: magnification 100×).

UHFUS examination of the first specific ROI (red circle) (Figure 2A) reveals a single fluid collection with a small pseudocyst. The fluid collection appears as a hypoechoic region with irregular shape, indicating the presence of fluid and inflammatory material within the abscess cavity. The surrounding structure appears more echogenic, potentially representing soft tissues and signs of inflammatory response in the adjacent tissues. Inside the fluid collection, it is possible to identify a ballooned hair follicle or pseudocyst‐like structure^16^, ^17^ with hyperechoic edges and heterogeneous content (1.74 mm × 2.96 mm) (Figure 2B). Another ROI (white circle) (Figure 2A) was then positioned in the structures adjacent to the fluid collection, corresponding to clinically healthy tissue, revealing dilated hair follicles in the left groin area. The image reveals multiple hypoechoic regions corresponding to the dilated follicles, likely due to inflammation and obstruction. The surrounding tissues exhibit a more echogenic appearance, indicating possible inflammatory changes (Figure 2C).

#### Histological Examination

Histology reveals a dermal mixed inflammatory cell infiltrate extending into the subcutaneous tissue, characterized by a dense neutrophilic infiltration, interstitial edema, peripheral granulation tissue and fibrosis. Within the inflammatory infiltrate a dilated infundibulum/pseudocyst‐like structure is appreciated (Figure 2, D–I). Therefore, in addition to the fluid collection, it is possible to identify the beginning of a non‐epithelialized tunnel and a dilated infundibulum. This histological finding reflects the typical complexity of lesions in HS, even when, from a clinical perspective, the lesion appears as a simple abscess.

### Case 2

**Figure 3:**
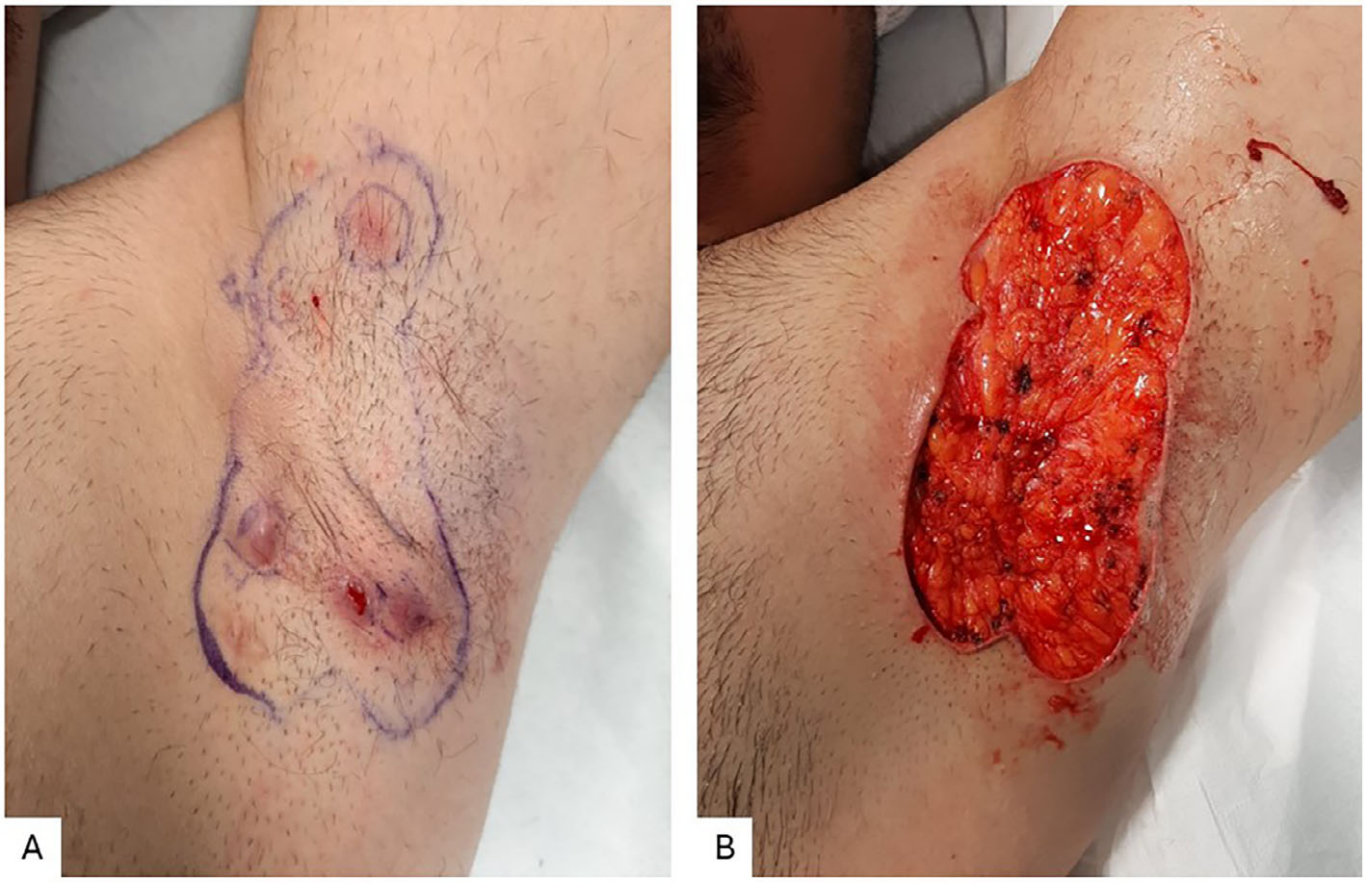
Case 2: Presurgical mapping (**A**) and postsurgical lesion after wide local excision of the left axilla (**B**).

Case 2 was a 35‐year‐old male patient with Hurley stage II HS for 10 years, exhibiting recurrent abscesses in both axillae, with scarring and tunnel separated by normal skin, currently undergoing treatment with Adalimumab 40 mg (administered bi‐weekly) and topical ultra‐potent corticosteroids. The patient was treated with wide excision of the area affected by the disease (Figure 3).

#### UHFUS Examination

**Figure 4:**
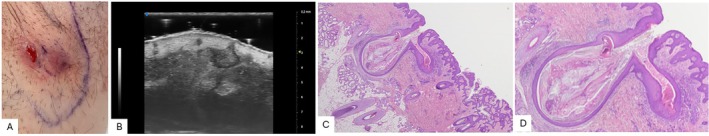
Case 2: Clinical examination of a nodule in the left axilla (**A**). UHFUS examination reveals a fluid collection with an elongated inclusion corresponding to the “drop‐shaped sign” or “donor sign” (**B**). Histological examination shows dilated a dilated hair follicle, connected to a tunnel and opening into the skin surface. A dense mixed inflammatory cell infiltrate embraces the structure (**C**: magnification 20×; **D**: magnification 40×).

UHFUS examination of the specific ROI reveals a fluid collection with an elongated inclusion located in the left armpit of a patient with HS (Figure 4A). The image reveals a hypoechoic area, consistent with a fluid collection, and a central inclusion with elongated shape with a well‐defined, hyperechoic border (3.04 mm × 0.73 mm). This structure corresponds to the “drop‐shaped sign” or “donor sign”^16^, ^17^ (Figure 4B).

#### Histological Examination

Histology demonstrates a superficial dilated hair follicle with a drop‐like shape, connected to a tunnel and opening into the skin surface. The cyst and the tunnel are embraced by a dense mixed inflammatory cell infiltrate (Figure 4, C and D).

### Case 3

**Figure 5:**
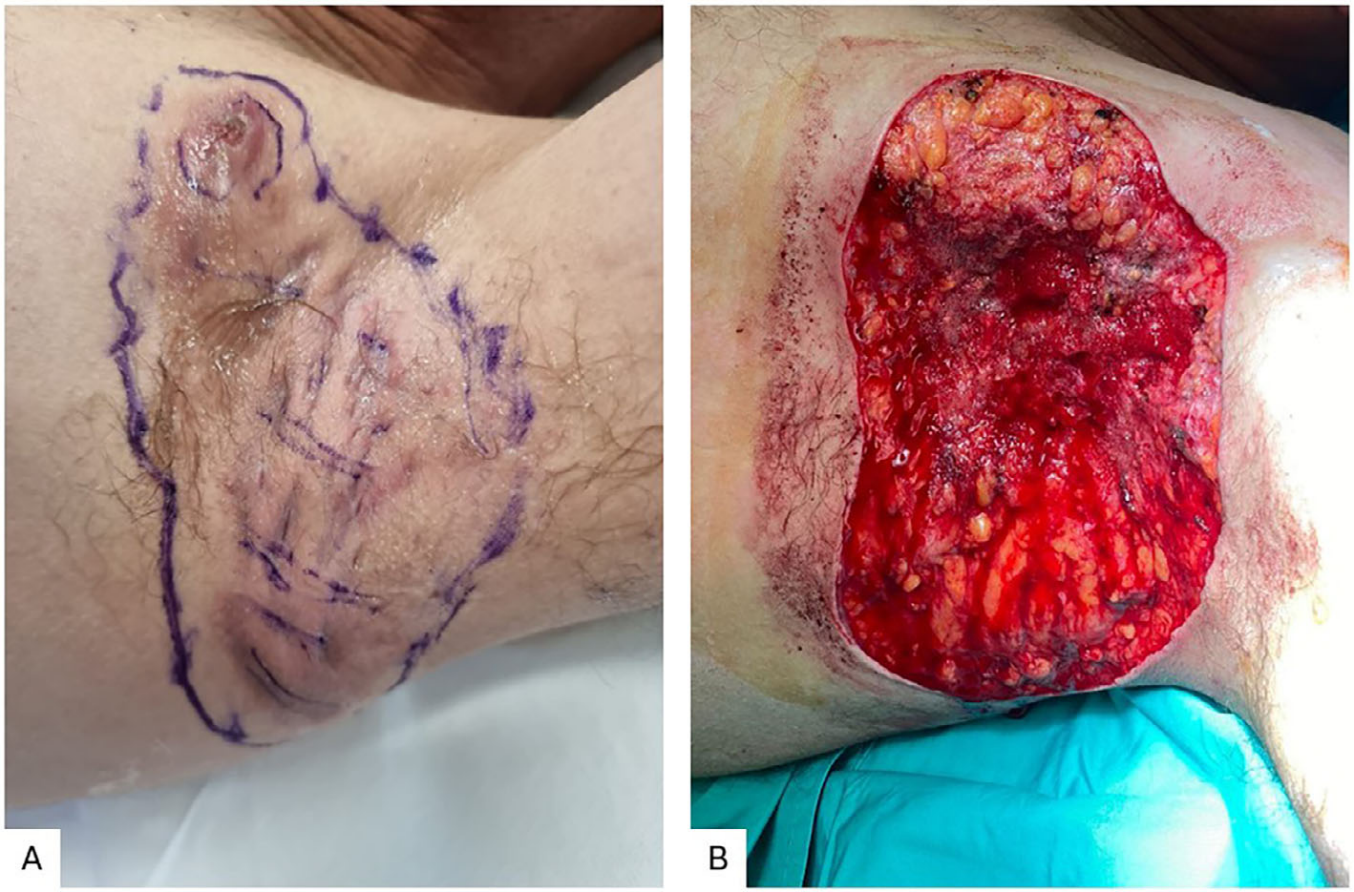
Case 3: Presurgical mapping (**A**) and postsurgical lesion after wide excision of the left axilla (**B**).

Case 3 was a 46‐year‐old male patient with Hurley stage III HS for 18 years, displaying recurrent lesions in both axillae and groin, with interconnecting tunnels without normal skin between lesions. He was treated with Adalimumab and localized corticosteroids. The patient underwent wide excision of the left axilla (Figure 5).

#### UHFUS Examination

**Figure 6:**
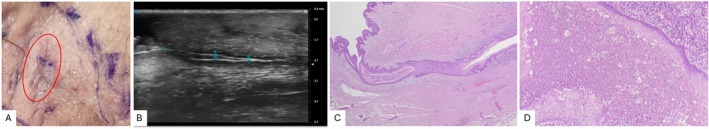
Case 3: Clinical examination of a draining fistula (red circle) in the left axilla (**A**). UHFUS examination reveals a tunnel (**B**). Histological examination shows a tunnel lined by stratified squamous epithelium extending from the skin surface through the reticular dermis (**C**: magnification 20×). The tunnel is filled with neutrophils and keratin debris and is surrounded by a dense chronic inflammatory cell infiltrate (**D**: magnification 100×).

The UHFUS examination of the ROI revealed a tunnel, localized in the dermis (Figure 6A). The tunnel structure appeared regular and linear, without significant expansions along its course, characterized by two hyperechoic band that delimited a hypoechoic structure. Tunnel length was ~2.7 cm with a diameter of 0.5 mm. The surrounding echogenicity showed relatively inhomogeneous hypoechoic tissue, suggesting a chronic inflammatory reaction associated with HS (Figure 6B).

#### Histological Examination

By histology, a tract lined by stratified squamous epithelium extending from the skin surface through the reticular dermis is evident. The tunnel is filled with keratin debris, numerous neutrophils, and amorphous material, and is surrounded by a dense chronic inflammatory cell infiltrate (Figure 6, C and D).

## Discussion

This study explored the correlation between UHFUS and histological features of HS lesions, providing insights into the diagnostic and staging potential of UHFUS in HS. By using a 70 MHz probe, we achieved a spatial resolution of 30 μm, effectively allowing for a near “in vivo” histopathological examination. This approach represents a significant advancement over traditional US frequencies, enabling more detailed visualization of microstructural changes in HS lesions and a closer approximation to histopathologic findings.

Our results highlight the advantages of UHFUS in detecting specific HS lesion characteristics, such as subclinical lesions, fluid collections, and tunnels, and their corresponding histopathologic features. In particular, we identified that UHFUS can delineate a fluid collection with internal inclusions and tunnels with high accuracy. These findings are consistent with existing literature, which has previously suggested the potential of UHFUS for identifying subclinical and early‐stage lesions that may not be detectable through clinical examination alone. The use of UHFUS as a complementary tool to clinical staging systems like the SOS‐HS and the mSOS‐HS allows for more precise disease assessment, which is critical for tailoring individualized treatment approaches and monitoring disease progression.^21^

In this study, we correlated specific UHFUS findings with histological features across different HS stages. In Case 1, UHFUS allowed us to clearly visualize a fluid collection and dilated hair follicles with surrounding inflammatory changes, while histology confirmed fluid accumulation and inflammatory cell infiltration. UHFUS examination identified an intra‐lesional iso‐hypoechoic, balloon‐shaped structure with a hyperechoic edge. This appearance has been previously described by Wortsman as “ballooning sign,” that represents the outcome of follicular dilation and seems to be the precursor stage of pseudocysts and fluid collections.^17^

In Case 2, we identified a well‐defined drop‐shaped inclusion within a fluid collection, which was corroborated by histological analysis. To date, the exact process of tunnel formation is not completely understood. However, correlation between US examination and histological examination reveals that draining tunnels represent an invagination of the epidermal tissue. The elongated structure within the fluid collection could therefore represent the beginning of microtunnel formation through epidermal invagination. As suggested in literature, in cases where drop‐shaped structures form, rupture of the hair follicle may lead to the formation of micro‐tunnels. This process could progress into tunnelization of the lesion or the development of fluid collections, potentially resulting in tunnel or pseudocyst formation.^16^, ^17^ As described by Kurzen et al the epithelial lining of the draining tunnels in HS is heterogeneous and comprises different types (I–III) of pathologically altered stratified squamous epithelia. However, in all three cases presented here these three types were not sharply defined, since transitional stages were commonly found (e.g., cornifying epithelium but inflamed): this reflects the dynamicity in the evolution of the disease.^22^ Finally, in Case 3, UHFUS delineated a tunnel structure within the dermis, showing linear hyperechoic bands surrounding a hypoechoic structure, which histology confirmed as a tunnel characteristic of chronic HS. The linear banded figures that clinically correspond to draining tunnels represent a section of an invagination of the epidermal tissue in the absence of signs of fibrosis and surrounded by a thin layer of inflammatory cells. The use of an US scanner with a 70 MHz probe allows to reveal that the two hyper‐echogenic bands bordering the tunnel are not indicative of fibrosis but rather represent keratinized epithelium. These findings align with previous studies that have demonstrated the utility of UHFUS for differentiating tissue structures and inflammatory patterns in HS lesions.^3^, ^15^ Grand et al in 2021 described the tunnels with a 10 to 22 MHz probe as hypoechoic/anechoic bands bordered by hyperechoic bands corresponding to the epithelialized tunnels visible on histology. The hyperechoic bands of the keratinized tunnels had higher echogenicity than the surrounding fibrosis that sometimes appears as a linear hyperechoic band on ultrasound examination.^19^ Moreover, histological and UHFUS comparisons show that at the level of the histological sections there is an optically empty cavity between the two invaginated epithelium which, on the other hand, appears hypoechoic in ultrasound images. This finding could testify how the optically empty cavity can be entirely occupied by liquid (like a “film”) of a possible inflammatory nature that acts as an interface for the visualization of the underlying layers since if it were formed by air, it would not have allowed the passage of ultrasound and the generation of shadow cones. This evidence could confirm the idea proposed by Navrazhina et al in 2021, according to which HS tunnels show higher infiltration of inflammatory cells than the overlying epidermis, exhibiting increased levels of T cells, dendritic cells, and neutrophils and higher expression of pro‐inflammatory cytokines.^14^

However, the study highlights some challenges in correlating UHFUS with histopathology in HS. For instance, while UHFUS provides high‐resolution images, translating these images to exact histological parameters remains complex. This discrepancy is primarily due to inherent limitations in correlating in vivo imaging with ex vivo histological samples; these include factors such as sampling constraints, tissue shrinkage during processing, loss of anatomical orientation after excision, and the dynamic nature of ultrasound compared with static histological sections. Furthermore, small sample size of the present study limits the generalizability of our findings. Future studies involving a larger patient cohort are needed to establish more standardized UHFUS parameters that could reliably correlate with histopathological findings.

The potential of UHFUS to assess tunnel formation in HS could serve as a biomarker for monitoring disease activity and treatment response, especially in patients on biological therapy. Krajewski et al demonstrated that patients with the “railway sign” at baseline showed a poorer response to biologic therapy, with complete healing in only 2.9 and 4.4% at weeks 12 and 24, compared with 64.7 and 88.2% in patients without this sign.^23^ In several cases, UHFUS revealed the presence of subclinical (“occult”) tunnels in areas of skin that appeared clinically uninvolved. These tunnels were typically located adjacent to active lesions but occasionally extended into seemingly healthy perilesional skin, highlighting the importance of imaging in the pre‐operative mapping of disease extent. Despite this, surgical excision often required broad margins, as microtunnels were frequently interconnected with deeper and clinically evident tunnels. Further studies might evaluate if biologics can avoid microtunnel progression.^24^

## Conclusions

In conclusion, our findings support the use of UHFUS as a valuable diagnostic and monitoring tool in HS, offering a non‐invasive method for assessing disease severity and guiding therapeutic decisions. The correlation between UHFUS and histological features of HS lesions suggests that UHFUS could serve as a reliable biomarker for disease staging and treatment efficacy. Further research is needed to validate these results in larger patient populations and to refine UHFUS protocols for HS to improve diagnostic accuracy and clinical utility.

## Data Availability

The data that support the findings of this study are available on request from the corresponding author. The data are not publicly available due to privacy or ethical restrictions.
